# Supplementary material for: Naturally acquired IgG responses to Plasmodium falciparum do not target the conserved termini of the malaria vaccine candidate Merozoite Surface Protein 2
Source: Front Immunol. 2024 Dec 9;15:1501700. doi: 10.3389/fimmu.2024.1501700 (PMC11663719; doi:10.3389/fimmu.2024.1501700)
Supplement: Supplementary file 1 [file DataSheet1.docx]

Supplementary Material

# Supplementary Data

# Supplementary Figures and Tables

## Supplementary Figures

**Supplementary Figure 1. Location of Nyamisati, Tanzania and reported country of infection for returning travelers with *Plasmodium falciparum* malaria.** (**A**) Travelers Cohort, where shades of red indicate the number of individuals returning from the country of infection (self-reported). (**B**) Location of the rural village of Nyamisati, which is within the Pwani region of the United Republic of Tanzania. Both surveys were started during the beginning of the long rains, which occur March through June. *P. falciparum* parasite rates in 1994 and 1999 were approximately 73% and 66%, respectively. Figure created in Datawrapper.

**Supplementary Figure 2. Conservation and consensus of expressed recombinant MSP2 protein sequences.** MAFFT alignment of amino acid sequences for the native form of the four MSP2 variants engineered and recombinantly expressed for this study, in order: MSP2-FC27 2, MSP2-FC27 1, MSP2-IC 1, MSP2-IC 2 (**A**) in addition to two reference MSP2 sequences from 3D7 and Dd2 *P. falciparum* strains; colored by percentage identity. Normalized, Valdar consensus score of the four expressed protein sequences and two reference sequences (**B**). Alignment performed in Galaxy and visualized in Jalview.

**Supplementary Figure 3. Distribution of IgG responses by ELISA is non-normal**. QQ plot of residuals for IgG antibody responses in normalized OD, for Travelers (**A**) and Tanzania samples (**B**).

**Supplementary Figure 4. IgG responses of autologous plasma towards MSP2 variants.** Plasma IgG from four travelers who were also carriers of the four expressed MSP2 variants used in this study, was evaluated for binding to the expressed full (**A**) or truncated (**B**) MSP2 variants, and conserved terminal peptides derived from the antigen (**C**). Dashed lines in (**A,B**) indicate mean cutoff for the shown variants. Dotted and dashed lines in (**C**) indicate mean cutoff for C- and N terminus peptides, respectively.

**Supplementary Figure 5. Detected IgG binding of all tested plasmas to full and truncated versions of four MSP2 variants as well as peptides from the conserved N- and C- termini.** A binary response matrix was created after applying a negative threshold for each cohort and each of ten tested antigens, for primary infected and previously exposed Travelers (**A**), Tanzania 1994 children and adults (**B**), and Tanzania 1999 children and adults (**C**). Plasma samples whose IgG responses to a specific antigen passed the negative cutoff are indicated by colored tiles while those that did not pass the cutoff for the antigen are indicated by white tiles.

**Supplementary Figure 6. Correlation of IgG responses to full and truncated MSP2 variants.** IgG responses in normalized OD values towards full MSP2 were plotted against those towards truncated MSP2 variants for primary infected and previously exposed travelers (**A**), Tanzania 1994 children and adults (**B**), and Tanzania 1999 children and adults (**C**). Correlation was estimated using Spearman rank correlation. Each color represents a different individual; therefore, every plot displays four data points of the same color corresponding to each of the four MSP2 variants tested.

**Supplementary Figure 7. IgG responses towards different MSP2 antigenic variants do not have a correlation with age.** IgG responses against full (**A**) and truncated (**B**) MSP2 variants and against conserved termini peptides (**E**), were plotted against age for individuals from Tanzania 1994. IgG responses against full (**C**) and truncated (**D**) MSP2 variants and against conserved termini peptides (**F**), were also plotted against age for individuals from Tanzania 1999. A Spearman rank correlation for each age group was fitted to sample data from all three sets. Sample data points are colored by survey and age group.

**Supplementary Figure 8. IgG responses towards full and truncated recombinant constructs, as well as conserved MSP2 termini according to microscopy-positivity and fever status.** IgG responses towards full (**A**) or Truncated (**B**) constructs of MSP2, as well as towards conserved termini peptides (**C**) were compared for Tanzanian children who were microscopy-negative or microscopy-positive for *P. falciparum* at the time of sampling. IgG responses towards full (**D**) or truncated (**E**) constructs, and conserved termini peptides (**F**) were also compared for Tanzanian adults who were microscopy-negative or -positive. IgG responses towards full (**G**) or truncated (**H**) constructs, and conserved termini peptides (**I**) were also compared between Tanzanian individuals who presented in a febrile state or not at the time of sampling. No adults had recorded fever and were not included in the comparison. Statistical significance by unpaired Wilcoxon test (* < 0.05, ** < 0.01).

**Supplementary Figure 9.** MSP2 peptide array setup schematic. The array consists of 128 13-mer MSP2 peptides, with 12 amino acid overlap giving single amino acid resolution. Included on each array were also positive control peptides from influenza and polioviruses. Figure created in BioRender.

**2.2 Supplementary Tables**

**Supplementary Table 1.** Primer and probe sequences used for *Plasmodium* species identification by qPCR, *msp2* amplification and barcoding for CCS, as well as size variant calling on *msp2* sequencing reads by *in silico* PCR.

| **Purpose and primer/probe name** | **Primer sequence (5’ → 3’)** |
| --- | --- |
| ***msp2* amplification and barcoding for CCS** **(Plaza *et al*, 2023)** | |
| 1^st^ reaction forward primer (msp2_fw) | ATGAAGGTAATTAAAACATTGTCTATTATA |
| 1st reaction reverse primer (msp2_rv) | TTATATGAATATGGCAAAAGATAAAACAA |
| 2^nd^ reaction forward primer | barcodes1001-to-1020-AATTTCTTTATTTTTGTTACC |
| 2^nd^ reaction reverse primer | barcodes1031-to-1050-GTGTTGCTGAAATTAAAAC |
| **Oligos for *msp2* family- and size variant-calling by *in silico* PCR** | |
| ICF1 | AGAAGTATGGCAGAAAGTAAGCCTCCTACT |
| ICF2 | AGAAGTATGGCAGAAAGTAATCCTCCTACT |
| ICF3 | AGAAGTATGGCAGAAAGTAAGCCTTCTACT |
| ICF4 | AGAAGTATGGCAGAAAGTAATCCTTCTACT |
| ICF_ML01 | AGAAGTATGGAAGAAAGTAATCCTCCTACT |
| ICF_GN01 | AGAAGTATGGCAGTAAGTAATCCTTCTACT |
| ICF_IT | AGAAGTATGACAGAAAGTAATCCTCCTACT |
| ICF_SD01 | AGAAGTATGTCAGAAAGTAAGCCTCCTACT |
| ICF_TG01 | AGAAGTATGACAGAAAGTAAGCCTCCTACT |
| ICF_GB4 | AGAAGTATGGCAGAAAGTAAGACTCCTACT |
| ICR | GATTGTAATTCGGGGGATTCAGTTTGTTCG |
| FC27F1 | AATACTAAGAGTGTAGGTGCAAATGCTCCA |
| FC27F2 | AATACTAAGAGTGTAGGTGCAGATGCTCCA |
| FC27F_KE01 | ACTACTAATAGTGTAGATGCAAATGCTCCA |
| FC27F_Dd2 | AATACTACTAGTGTAGGTGCAAATGCTCCA |
| FC27F_CD01_SN01 | AATACTAATAGTGTAGGTGCAGATGCTCCA |
| FC27F_HB3 | AATACTAAGAGTGTAGGTGCAAATGCTCCA |
| FC27R | TTTTATTTGGTGCATTGCCAGAACTTGAAC |

**Supplementary Table 2.** **MSP2 variants sequenced in isolates from *P. falciparum*-positive travelers and expressed as recombinant proteins.** Protein sequences, translated from CCS reads, are shown in the native form (As expressed by the parasite). The sequences shown here include native signal peptides, GPI anchoring signals and predicted N-glycosylation sites.

| **Isolate (Reported Country of Origin)** | **Size variant (bp)** | **Reads / % Sample Coverage** | **Protein Sequence** |
| --- | --- | --- | --- |
| MSP2-FC27 1 (Gambia) | 292 | 25 / 72.0 | MKVIKTLSIINFFIFVTFNIKNESKYSNTFINN AYNMSIRRSMANEGSNTTSVGANAPNADT IANGSQSSTNSASTSTTNNGESQTTTPTAAD TPTATKSNSPSPPITTTKSNSPSPPITTTKSNS PSPPITTTESSSSGNAPNKTDGKGEESEKQN ELNESTEEGPKAPQEPQTAENENPAAPENK GTGQHGHMHGSRNNHPQNTSDSQKECTD GNKENCGAATSLLNNSSNIASINKFVVLISA TLVLSFAIFI |
| MSP2-IC 1 (South-East Asia) | 575 | 238 / 97.1 | MKVIKTLSIINFFIFVTFNIKNESKYSNTFINN AYNMSIRRSMEESNPSTGAGGSGSAGGSGS AGGSGSAGGSGSAGGSGSAGGSGSAGGSG SAGGSGSAGGSGSAGGSGSAGSGDGNGAN PGADAERSPSTPATTTTTTTTNDAEASTSTS SENPNHNNAETNPKGKGEVQKPNQANKET QNNSNVQQDSQTKSNVPPTQDADTKSPTA QPEQAENSAPTAEQTESPELQSAPENKGTG QHGHMHGSRNNHPQNTSDSQKECTDGNK ENCGAATSLLSNSSNIASINKFVVLISATLV LSFAIFI |
| MSP2-IC 2 (Ghana) | 707 | 77 / 80.5 | MKVIKTLSIINFFIFVTFNIKNESKYSNTFINN AYNMSIRRSMAESNPSTGAGGSGSAGGSA GGSAGGSAGGSAGGSAGGSAGGSAGGSAG GSAGGSAGGSAGGSAGGSAGGSAGGSAGG SAGGSAGGSAGGSAGGSAGGSAGGSAGGS AGGSAGGSAGGSAGSGDGNGANPGADAE GSSSTPATTTTTTTTTTTNDAEASTSTSSEN PKGKGEVQKPNQANKETQNNSNVQQDSQ TKSNVPRTQDADTKSPTAQPEQAENSAPTA EQTESPELQSAPENKGTGQHGHMHGSRNN HPQNTSDSQKECTDGNKENCGAATSLLNN SSNIASINKFVVLISATLVLSFAIFI |
| MSP2-FC27 2 (Kenya) | 376 | 571 / 95.1 | MKVIKTLSIINFFIFVTFNIKNESKYSNTFINN AYNMAIRRSMANKGSNTNSVGANAPNAD TIASGSQRSTNSASTSTTNNGESQTTTPTAA DTIASGSQRSTNSASTSTTNNGESQTTTPTA ADTIASGSQRSTNSASTSTTNNGESQTTTPT AADTPTATESSSSGNAPNKADGKGEESEKQ NELNESTEEGPKAPQEPQTAENENPAAPEN KGTGQHGHMHGSRNNHPQNTADSQKECT DGNKENCGAATSLLNNAANIASINKFVVLI SATLVLSFAIFI |
