# Supplementary figures and images for: Naturally acquired IgG responses to Plasmodium falciparum do not target the conserved termini of the malaria vaccine candidate Merozoite Surface Protein 2
Source: Front Immunol. 2024 Dec 9;15:1501700. doi: 10.3389/fimmu.2024.1501700 (PMC11663719; doi:10.3389/fimmu.2024.1501700)

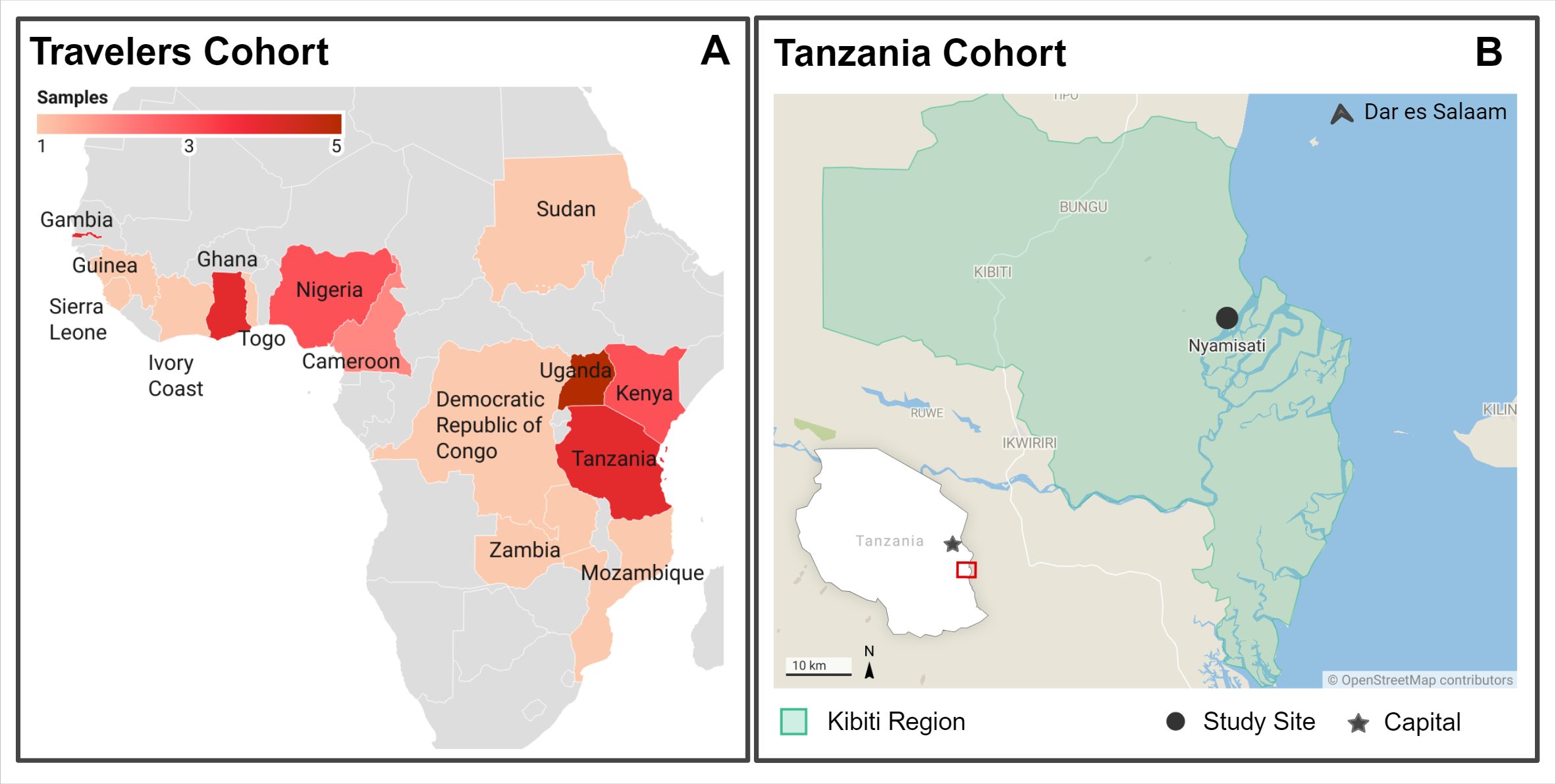

Supplement: Supplementary file 2 [file Image1.jpeg]

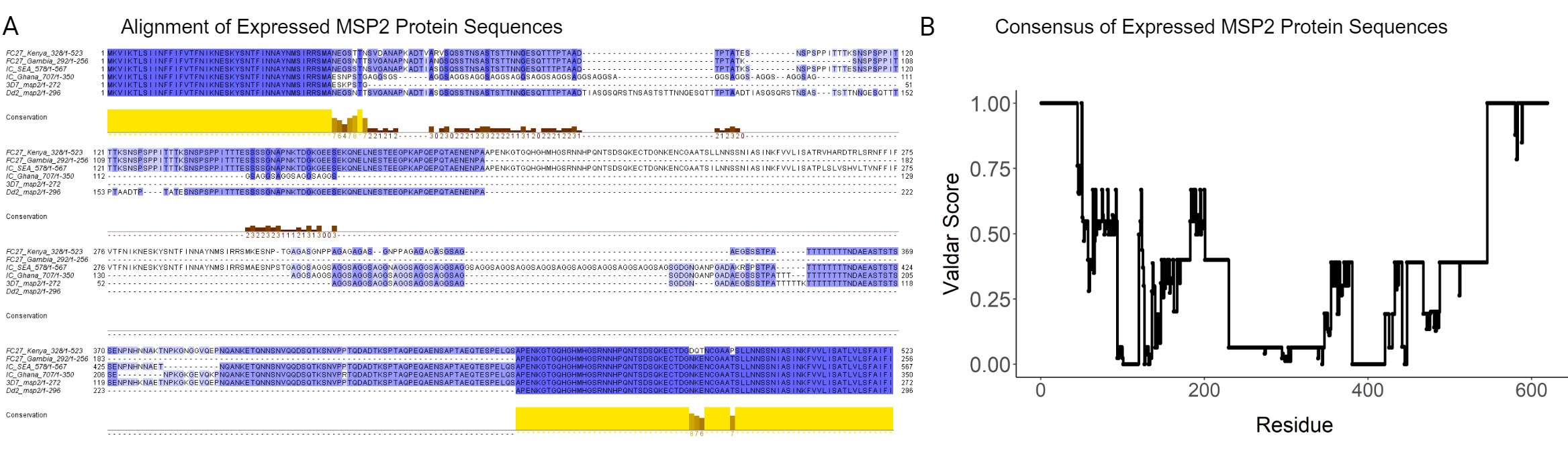

Supplement: Supplementary file 3 [file Image2.jpeg]

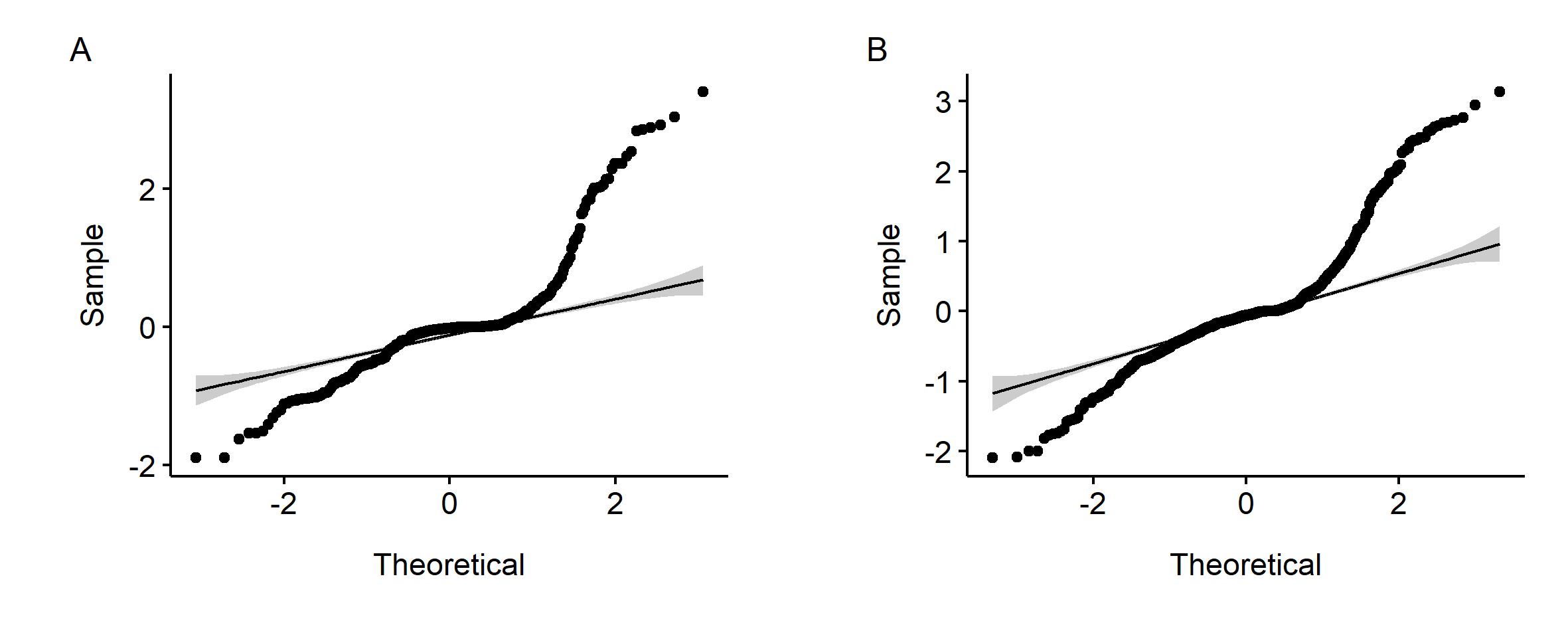

Supplement: Supplementary file 4 [file Image3.jpeg]

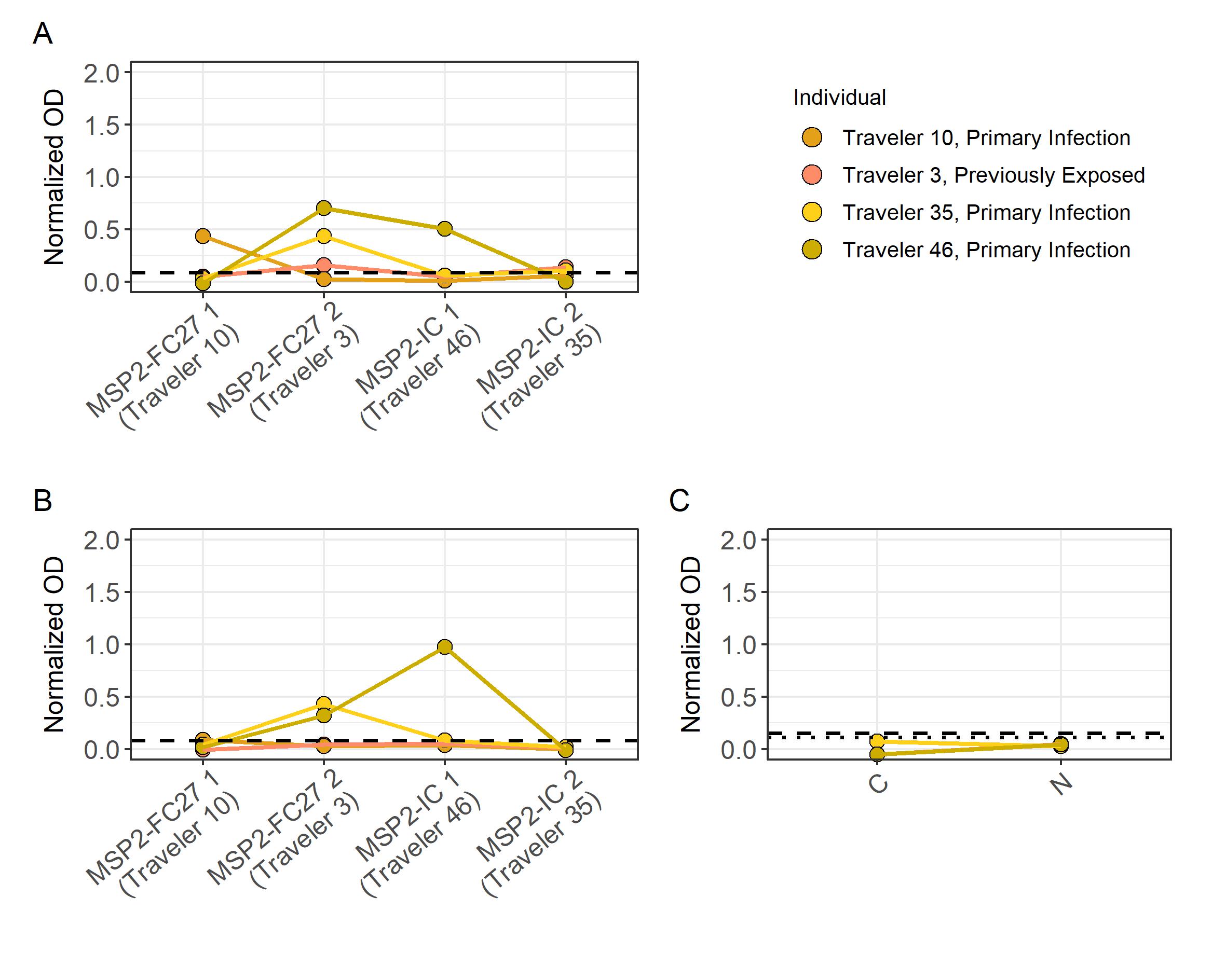

Supplement: Supplementary file 5 [file Image4.jpeg]

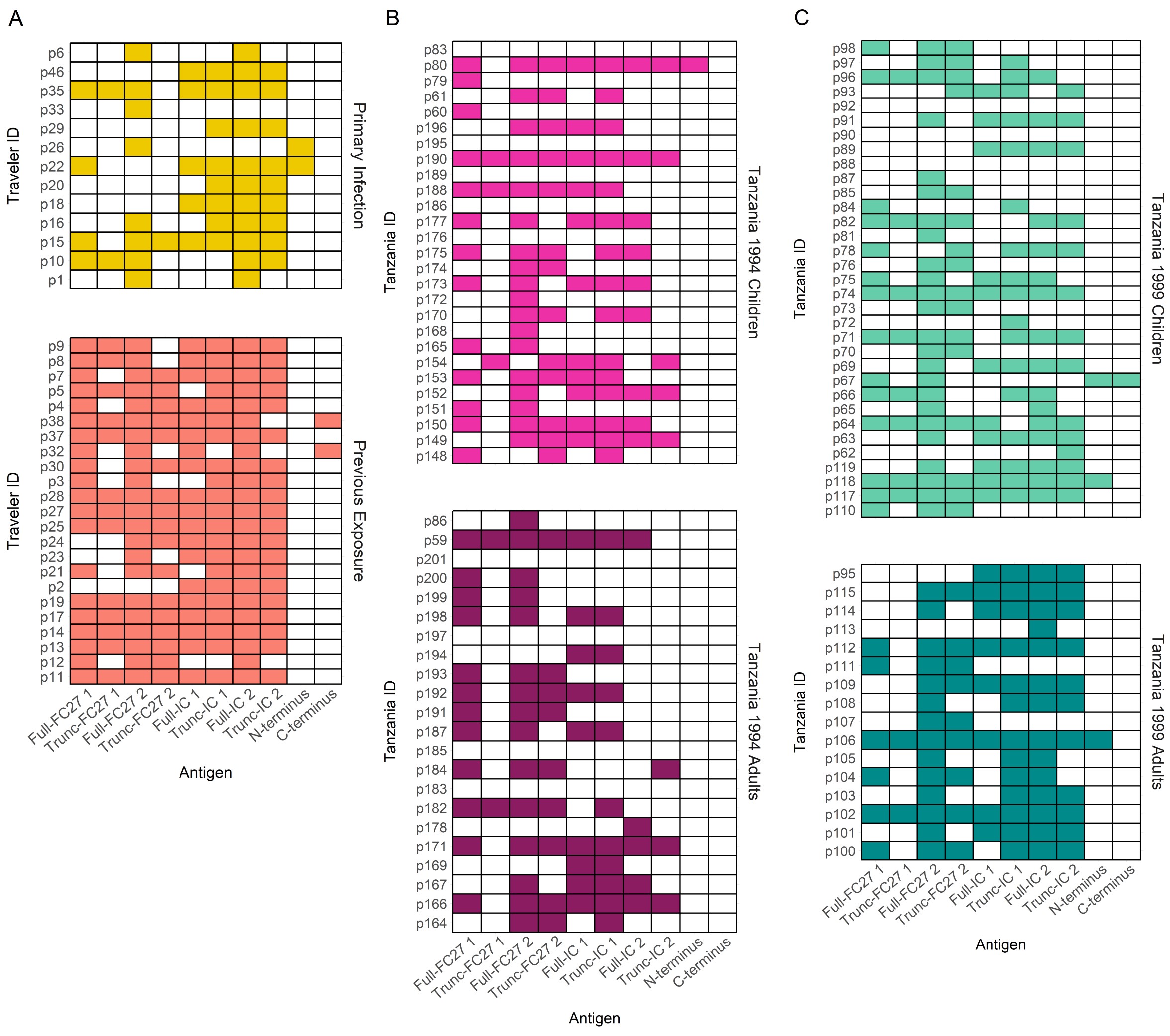

Supplement: Supplementary file 6 [file Image5.jpeg]

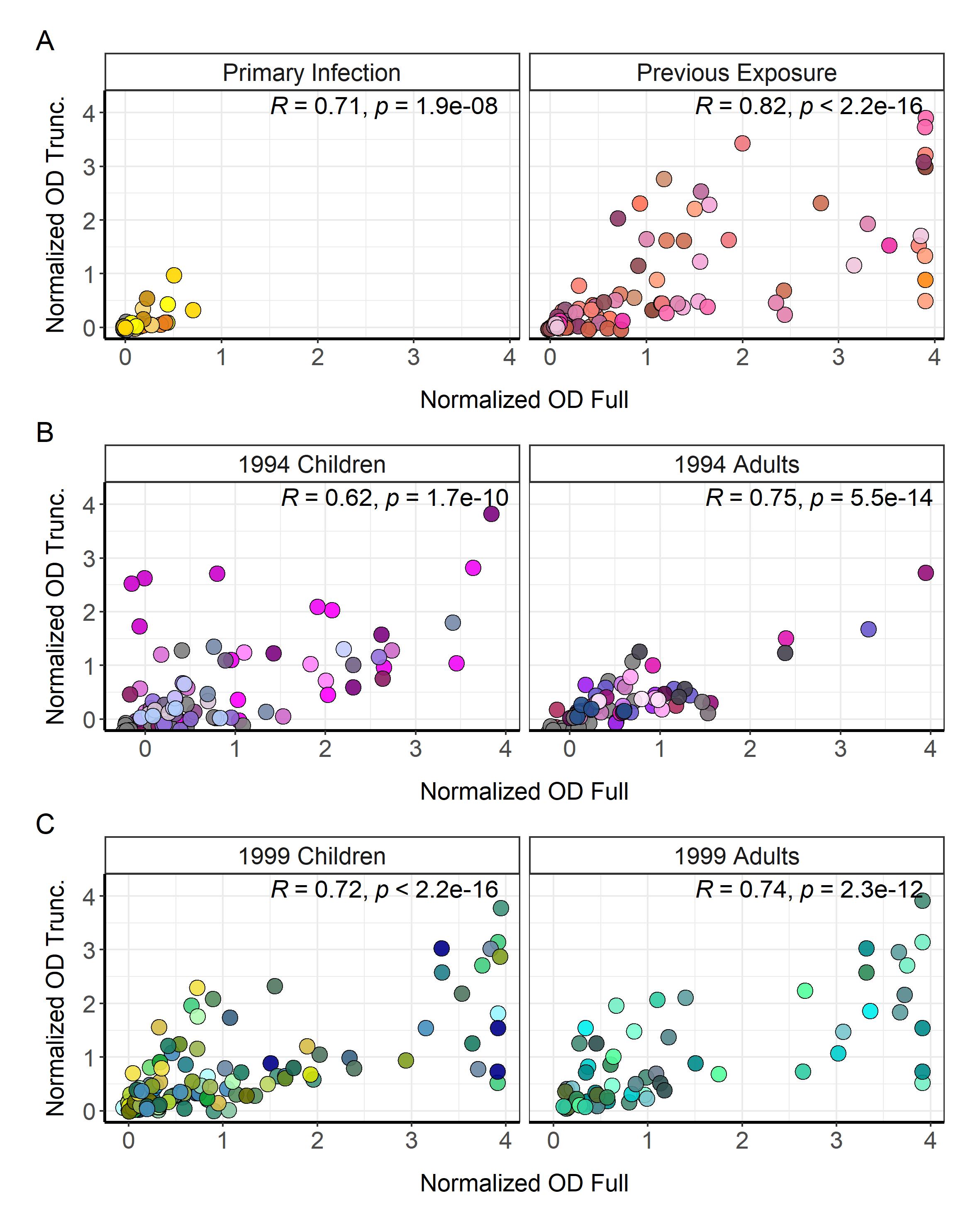

Supplement: Supplementary file 7 [file Image6.jpeg]

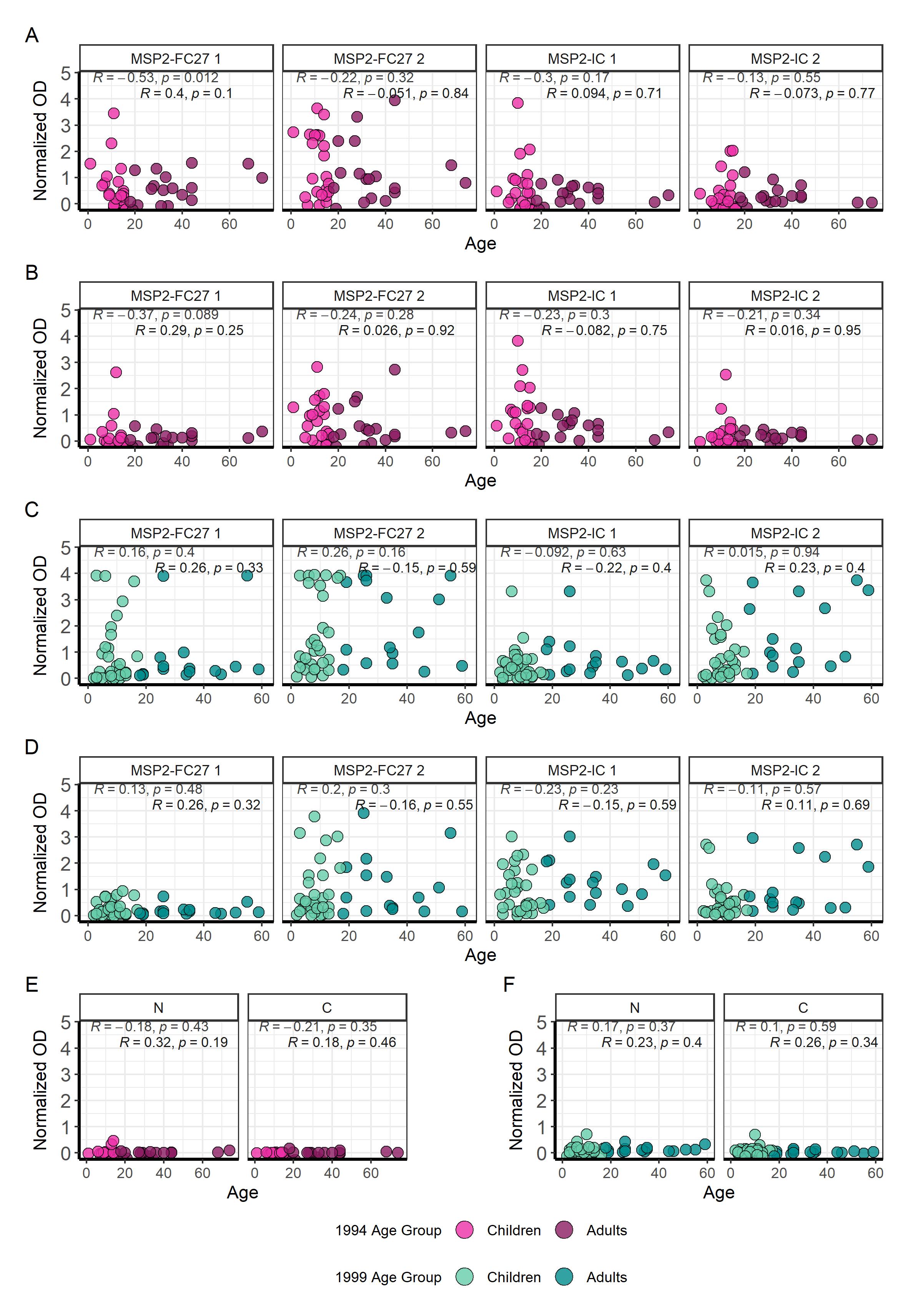

Supplement: Supplementary file 8 [file Image7.jpeg]

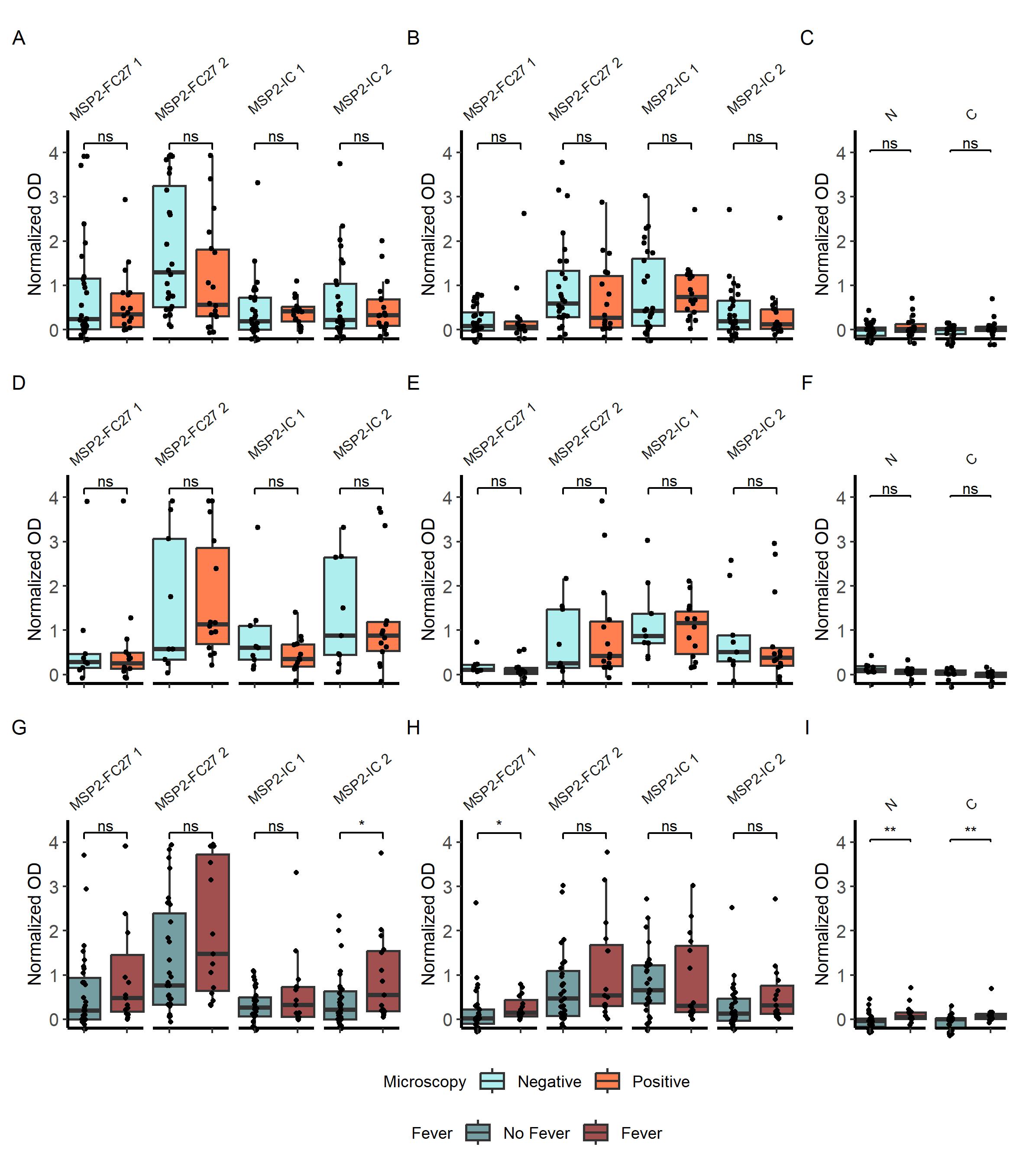

Supplement: Supplementary file 9 [file Image8.jpeg]

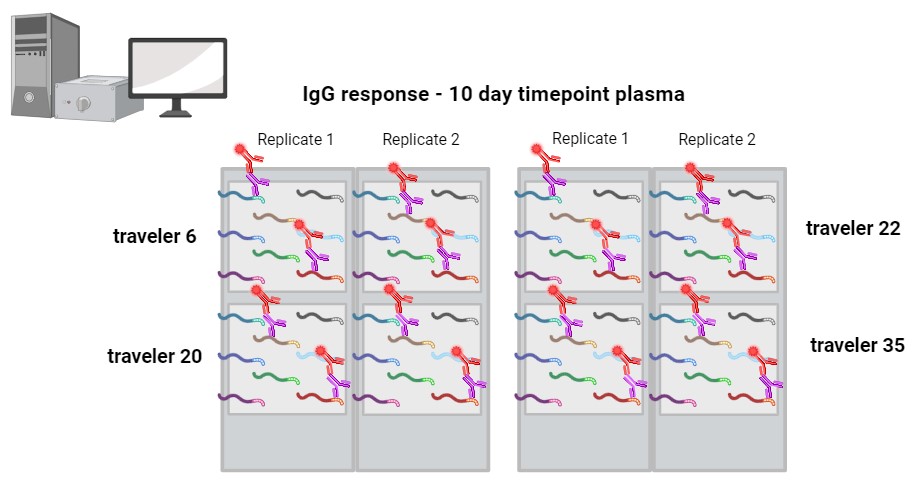

Supplement: Supplementary file 10 [file Image9.jpeg]
